# Diversity in the complexity of phosphate starvation transcriptomes among rice cultivars based on RNA-Seq profiles

**DOI:** 10.1007/s11103-013-0106-4

**Published:** 2013-07-16

**Authors:** Youko Oono, Yoshihiro Kawahara, Takayuki Yazawa, Hiroyuki Kanamori, Masato Kuramata, Harumi Yamagata, Satomi Hosokawa, Hiroshi Minami, Satoru Ishikawa, Jianzhong Wu, Baltazar Antonio, Hirokazu Handa, Takeshi Itoh, Takashi Matsumoto

**Affiliations:** 1 Plant Genome Research Unit, National Institute of Agrobiological Sciences, Kannondai 2-1-2, Tsukuba, Ibaraki 305-8602 Japan; 2 Bioinformatics Research Unit, National Institute of Agrobiological Sciences, Kannondai 2-1-2, Tsukuba, Ibaraki 305-8602 Japan; 3 Hitachi Government & Public Corporation System Engineering, Ltd, 2-4-18 Toyo, Koto-ku, Tokyo 135-8633 Japan; 4 Soil Environment Division, National Institute for Agro-Environmental Sciences, Tsukuba, Ibaraki 305-8604 Japan; 5 Department of Genome Informatics, Mitsubishi Space Software Co., Ltd., Takezono 1-6-1, Tsukuba, Ibaraki 305-0032 Japan; 6 Advanced Genomics Laboratory, National Institute of Agrobiological Sciences, Kannondai 2-1-2, Tsukuba, Ibaraki 305-8602 Japan; 7 Genome Resource Unit, National Institute of Agrobiological Sciences, Kannondai 2-1-2, Tsukuba, Ibaraki 305-8602 Japan; 8 Agriculture, Forestry and Fisheries Research Council, Ministry of Agriculture, Forestry and Fisheries, Kasumigaseki 1-2-1, Chiyoda-ku, Tokyo 100-8950 Japan

**Keywords:** Abiotic stress, Phosphate starvation, Phosphorus, Transcriptome, RNA-Seq, Rice

## Abstract

Rice has developed several morphological and physiological strategies to adapt to phosphate starvation in the soil. In order to elucidate the molecular basis of response to phosphate starvation, we performed mRNA sequencing of 4 rice cultivars with variation in growth response to Pi starvation as indicated by the shoot/root dry weight ratio. Approximately 254 million sequence reads were mapped onto the IRGSP-1.0 reference rice genome sequence and an average of about 5,000 transcripts from each cultivar were found to be responsive under phosphate starvation. Comparative analysis of the RNA-Seq profiles of the 4 cultivars revealed similarities as well as distinct differences in expression of these responsive transcripts. We elucidated a set of core responsive transcripts including annotated and unannotated transcripts commonly expressed in the 4 cultivars but with different levels of expression. De novo assembly of unmapped reads to the Nipponbare genome generated a set of sequence contigs representing potential new transcripts that may be involved in tolerance to phosphate starvation. This study can be used for identification of genes and gene networks associated with environmental stress and the development of novel strategies for improving tolerance to phosphate starvation in rice and other cereal crops.

**Electronic supplementary material:**

The online version of this article (doi:10.1007/s11103-013-0106-4) contains supplementary material, which is available to authorized users.

## Introduction

Phosphorus (P) is one of the essential macronutrient for growth and productivity of cereal crops but is also one of the least available in the soils. It has therefore become a major component of inorganic fertilizers used in modern agriculture to achieve high yield of various crops. In recent years, excessive application of phosphate (Pi) has become a major concern because this non-renewable element is continuously being depleted at an alarming rate (Raghothama 1999). As plants in general rely on P for many biological functions particularly for storage and transfer of energy, which are involved in almost all metabolic processes throughout growth and development, considerable research has focused on the physiological and biochemical mechanisms of adaptation to Pi starvation (Miura et al. 2005, Jiang et al. 2007), identification of genes that control Pi stress tolerance (Rubio et al. 2001, Bari et al. 2006, Gamuyao et al. 2012), and characterization of response to Pi stress at the genome level (Wasaki et al. 2003a; Misson et al. 2005; Oono et al. 2011).

Rice adapts to Pi stress with a wide range of morphological changes such as increased root proliferation and physiological changes associated with efficient P acquisition, transport and utilization. Genes involved in enhancing P acquisition efficiency from the soil, increasing its utilization efficiency via remobilization, and translocation from shoots to roots, all of which function to compensate for the adverse effects on metabolic processes that rely on high-energy Pi compounds have been analyzed (Huang et al. 2011). The roles of transcription factors such as PHR1 (Rubio et al. 2001), WRKY75 (Devaiah et al. 2007), OsPTF1 (Yi et al. 2005), as well as genes encoding high-affinity Pi transporters (Paszkowski et al. 2002), RNases (Bariola et al. 1994), acid phosphatases (Hur et al. 2010; Wang et al. 2011), and non-protein coding gene *IPS1* (Hou et al. 2005) have been the focus of studies aimed at elucidating the mechanism of tolerance to Pi stress. Additionally, genetic variation in terms of P uptake on Pi deficient soils has been pursued with the aim of identifying tolerant cultivars that can be used in breeding. Analysis of four distinct barley genotypes showed that genetic variation in P acquisition efficiency required optimization of utilization efficiency, which was correlated with the expression of low-affinity Pi transporters and *IPS1* (Huang et al. 2011). In rice, several cultivars were analyzed in terms of P content on Pi deficient soils (Wissuwa and Ae 2001). The quantitative trait locus for tolerance to Pi starvation has been identified from a cross between the *japonica* cultivar Nipponbare with low tolerance and the *indica* cultivar Kasalath with high tolerance, and the QTL was eventually transferred to a near-isogenic line (NIL) of Nipponbare that eventually showed much higher P content and grain yield than the sensitive cultivar Nipponbare (Wissuwa and Ae 2001).

Recent advances in structural and functional genomics strategies led to deeper analysis of response to Pi starvation at the transcriptome level. Wasaki et al. (2003a) used a microarray platform with 8,987 ESTs to characterize the gene expression profile of Pi deficient rice roots. This study led to the characterization of *OsPI1*, which shares some of the properties of *TPSI1/Mt4*, the Pi starvation inducible gene family that plays an important role in the early stages of adaptation to low Pi availability. Using the 22K microarray platform, the gene expression level under Pi starvation between Nipponbare and NIL6-4 carrying a major QTL for Pi starvation tolerance *Pup1*, was analyzed (Pariasca-Tanaka et al. 2009). Genes putatively associated with root cell wall loosening and root hair extension such as xyloglucan endotrans-glycosylases/hydrolases and NAD (P) H-dependent oxidoreductase showed higher expression in roots of tolerant NIL6-4. Analysis of gene expression profiles of two *indica* varieties, a low P-tolerant Zhongzao 18 and not so low P-tolerant Lagrue under low P stress showed that several genes involved in glycolysis and TCA cycle were upregulated during the early stages of low P treatment in roots of Zhongzao 18 but not in root of Lagrue (Li et al. 2010). These studies clearly showed genetic variation in response to Pi starvation. However, since only a limited number of associated genes with response to Pi starvation were characterized, the entire mechanism of Pi starvation tolerance at the molecular level is barely elucidated.

We therefore embarked on RNA-Seq analysis of four *Oryza sativa* cultivars to characterize the variation in the transcriptomes in response to Pi starvation and to provide an overview of the regulatory mechanisms associated with Pi stress tolerance in rice and other cereal crops. We analysed the transcriptome of a *japonica* cultivar Nipponbare with low tolerance to Pi stress, two *japonica* cultivars, namely, IAC 25 and Vary Lava 701 with relatively higher tolerance, and an *indica* cultivar Kasalath, which is known to be highly tolerant to Pi stress.

## Materials and methods

### Plant materials and growth evaluation

Seeds of the *japonica* cultivars Nipponbare, IAC 25 and Vary Lava 701, and *indica* cultivar Kasalath were germinated and grown by hydroponic culture in Yoshida nutrient medium which consisted of 1.425 mM NH_4_NO_3_, 0.323 mM NaH_2_PO_4_, 0.513 mM K_2_SO_4_, 0.998 mM CaCl_2_, 1.643 mM MgSO_4_, 0.009 mM MnCl_2_, 0.075 mM (NH_4_)_6_Mo_7_O_24_, 0.019 mM H_3_BO_3_, 0.155 mM CuSO_4_, 0.036 mM FeCl_3_, 0.070 mM citric acid, and 0.152 mM ZnSO_4_ (Yoshida et al. 1976). Two-week old seedlings were subjected to Pi starvation treatment by transferring in the same nutrient medium but with the Pi concentration reduced to 0.00323 mM NaH_2_PO_4_.

The total dry weight of root and shoot samples from seedlings grown in Pi deficient medium and from untreated control were measured at regular intervals. Additionally, the dry weights under an overabundant supply of Pi were also determined for comparative purposes using root and shoot samples from seedlings grown in nutrient medium containing 3.23 mM NaH_2_PO_4_. The total P content per plant and P concentration in 1 mg plant sample from Pi deficient medium and control were measured as described previously (Oono et al. 2011). The inorganic Pi content was determined by releasing the cellular content of cells in water through repeated freeze–thaw cycle, and quantification with the molybdate assay method (Ames 1966).

The samples used for RNA preparation were collected before the onset of stress treatment (0 d), and after 10 days (10 d) and 22 days (22 d) of growth in Pi deficient medium, frozen immediately in liquid nitrogen, and stored at −80 °C until extraction.

### Confirmation of expression by qRT-PCR

The expression of *IPS1* and other Pi starvation responsive genes in the root and shoot samples of the 4 rice cultivars was confirmed by quantitative RT-PCR (qRT-PCR) analysis using three technical replicates from one of the three biological replicates used for RNA-Seq analysis. Frozen root and shoot samples collected at 0, 10 and 22 d of Pi starvation treatment were grounded separately. Total RNA was extracted from those samples using the RNeasy Plant Kit (Qiagen, Hilden, Germany) and treated with DNase I (Takara, Shiga, Japan). The first-strand cDNA was synthesized using the Transcriptor First Strand cDNA synthesis kit (Roche, Basel, Switzerland) according to the manufacturer’s protocol. The resulting cDNAs were amplified in the LightCycler^®^ 480 system (Roche, Basel, Switzerland) using transcript-specific primers (Supplementary Table S1). The detection threshold cycle for each reaction was normalized using *Ubiquitin1* with 5′-CCAGGACAAGATGATCTGCC-3′ and 5′-AAGAAGCTGAAGCATCCAGC-3′ as primers.

### RNA-Seq analysis and identification of responsive transcripts

Total RNA from root and shoot samples was extracted and processed for construction of cDNA libraries using the TruSeq™ RNA sample preparation kit. We constructed a total of 48 cDNA libraries corresponding to root and shoot of the 4 cultivars at 0 and 22 d of Pi starvation treatment with three biological replicates for each sample. Sequencing was performed in the Illumina Genome Analyzer IIx as described previously (Oono et al. 2011). The sequence reads filtered by CASAVA (ver. 1.8) were removed using a customized Java program. Stretches of low quality bases at both sides of reads were trimmed using a customized C program (Q value <15). Adapter sequences were removed using cutadapt version 1.0 (http://code.google.com/p/cutadapt/) with default parameters. All reads were aligned to rice rRNA genes using Bowtie version 0.12.7 (http://bowtie-bio.sourceforge.net/index.shtml) with parameters (-q–threads 2–sam–un) to remove reads derived from rRNA molecules. After pre-processing the Illumina reads, the transcript structures were reconstructed using a series of programs, namely, Bowtie version 0.12.7 for short-read mapping (Langmead et al. 2009), TopHat version 1.4.1 for defining exon–intron junctions (Trapnell et al. 2009), and Cufflinks version 1.3.0 for gene structure predictions (Trapnell et al. 2010). For TopHat, the Os-Nipponbare-Reference-IRGSP-1.0 (IRGSP-1.0) pseudomolecules (http://rapdb.dna.affrc.go.jp/) were used as the reference sequences with the following options: segment-length 20, segment-mismatches 1, min-intron-length 30, max-intron 6000, max-multihits 40, no-closure-search, min-coverage-intron 30, max-coverage-intron 6000, min-segment-intron 30, max-segment-intron 6000, coverage-search, num-threads 2. All reads that could not be aligned to the IRGSP-1.0 reference genome sequence were separately analysed as described below. The expression level for each transcript was calculated as reads per kilobase of exon model per million mapped (RPKM) values based on the number of uniquely mapped reads that completely overlap with the exonic regions, using at least 2 replicates with correlation coefficient of >0.92 in each library. To detect transcripts expressed as a response to Pi starvation, G-test was performed on the read count of transcripts obtained from root and shoot at 0 and 22 d of stress treatment. The number of mapped reads on a given transcript and those on other regions for two stages were used as variables in 2 × 2 contingency tables for each test. All *p*-values were corrected with false discovery rate (FDR) of 0.1 % using the R package version 2.14.2 and in-house Perl scripts (Benjamini and Hochberg 1995). The resulting RNA-Seq data have been deposited to the DNA Data Bank of Japan (DDBJ) sequence read archive under the accession number DRA000685.

### Venn diagram, hierarchical clustering and GO enrichment analysis

The upregulated and downregulated transcripts in the 4 cultivars were used for Venn diagram analysis using R base package version 2.14.0 and in-house Perl scripts. The commonly upregulated transcripts in root and shoot among the 4 cultivars were used for hierarchical clustering analysis. We used the heatmap.2 in the R package gplots (ver. 2.11.0) to perform clustering analyses of transcripts. The Z scores were used to compare significant changes in gene expression including fold changes. A GO term was assigned to each transcript based on the GO annotations for biological process, molecular function and cellular component in RAP-DB. GO enrichment was evaluated by Fisher’s exact test with a FDR threshold of 5 % for responsive transcripts in the biological process category which overlapped among the 4 cultivars. The results were plotted as −log10 of FDR values in a heatmap.

### Transcript assembly of unaligned reads

The sequence reads from each cultivar that could not be aligned to the IRGSP-1.0 genome sequence were assembled into contigs of various k-mer sizes (k = 21 to k = 51) using various options for Velvet version 1.2.03 (parameter for velveth; -fastq -short’, for velvetg; -read_trkg yes) and Oases version 0.2.05 (defaults). The resulting contigs were merged into a final assembly with parameters ‘27 -long’ (for velveth), ‘-read_trkg yes -conserveLong yes’ (for velvetg) and ‘-merge yes’ (for oases). Redundant contigs were removed using the cd-hit-est version 4.5.4 with default parameters. To retrieve genotype specific contigs, the contigs were mapped against the IRGSP-1.0 genome sequence by Blat version v.34 resulting in the removal of contigs with more than 50 % coverage. To infer the function of contigs, a Blastx search against the NCBI RefSeq and SwissProt databases were performed using E-value of 1E − 10 as cutoff threshold. For RefSeq, only transcripts or protein records with status of ‘validated’ or ‘reviewed’ were used. Furthermore, only contigs with hit to proteins of land plant species were retained to eliminate contaminations. Lastly, contigs from IAC 25, Vary Lava 701 and Kasalath similar to Nipponbare were eliminated and the remaining contigs were identified as genotype specific. The reads used for *de novo* assembly were aligned back to the contigs and the RPKM values were calculated as described above.

## Results

### Changes in plant growth induced by Pi starvation

The rice seedlings of the 4 cultivars subjected to Pi starvation (−P) stress began to show difference in growth from the control (+P) after 10 days of treatment. From then on, the difference in growth gradually became more evident, and at 22 days of Pi starvation, there were significant differences between the treated and non-treated plants in dry weight, total P content, and P concentration of both roots and shoots. The 4 cultivars showed variation in growth response to −P as shown by the relative values of dry weight, total P content and P concentration calculated with the corresponding values in +P as control (Table 1). The average dry weight of shoot per plant after 22 days under −P was almost the same as +P in IAC 25 and Vary Lava 701 (Supplementary Fig. S2a). On the other hand, the average dry weight of root per plant gradually increased in IAC 25, Vary Lava 701 and Kasalath after 10 days under −P as compared to +P (Supplementary Fig. S2b). However, under overabundant supply of Pi (++P), the 4 cultivars showed a decrease in both shoot and root dry weights (Supplementary Fig. S2a, S2b), suggesting a possible toxic effect of excess Pi supply during early growth stages. The shoot/root dry weight ratio was higher for Nipponbare (4.2 ± 0.15) as compared to IAC 25 (3.4 ± 0.08), Vary Lava 701 (3.7 ± 0.15) and Kasalath (2.9 ± 0.2) after 22 days under −P (Supplementary Fig. S2c). These three cultivars were classified as tolerant and more adaptable to P starvation. The shoot/root dry weight ratio resulted from increased root growth rate after 22 days under −P with IAC 25, Vary Lava 701 and Kasalath showing higher relative values than Nipponbare (Supplementary Fig. S2b, S2c). In contrast, the shoot/root dry weight ratio after 22 days under ++P with IAC 25 and Vary Lava 701 showed higher relative values as compared to Nipponbare.

**Table 1:** Effect of Pi starvation on dry weight, total P content and P concentration relative to control of rice seedlings after 10 and 22 days in −P medium

	Relative value (Pi starvation/control, control = 100)^a^							
	Nipponbare		IAC 25		Vary Lava 701		Kasalath	
	10 d	22 d	10 d	22 d	10 d	22 d	10 d	22 d
Root weight	88.4	112.9	131.9	173.7	116.5	134.6	96.1	135.8
Shoot weight	63.1	70.7	100.1	96.1	103.2	86.6	76.4	64.2
Total P content of root	60.0	14.6	80.2	21.1	49.9	23.5	41.4	17.0
Total P content of shoot	55.2	17.5	52.4	14.9	45.0	20.0	37.4	15.6
P concentration of root	82.2	12.0	46.6	6.9	40.7	13.6	46.2	10.3
P concentration of shoot	134.0	34.7	52.2	16.0	44.2	27.2	64.2	39.8

^a^Based on measurements for dry weight as mg/plant, total P content as ug P/plant, P concentration as nmol/mg dry weight. The relative value was calculated based on the average values for each treatment relative to control. The SD for the average values are indicated in Supplementary Figures S2 and S3. Control = +P treatment at 10 or 22 d

The total P content of root and shoot per plant decreased from 0 d until 10 d under −P and remained at this level at 22 d in all cultivars (Supplementary Fig. S3a). In contrast, the P concentration gradually decreased from 0 d until 22 d under −P in all cultivars (Supplementary Fig. S3b). Among the 4 cultivars, relative P content of IAC 25 and Kasalath in root was higher than Nipponbare whereas relative P concentration was lower than Nipponbare (Table 1). Furthermore, although the P content of Vary Lava 701 in root was higher than Nipponbare, the P concentration was higher than Nipponbare.

**Fig. 1:**
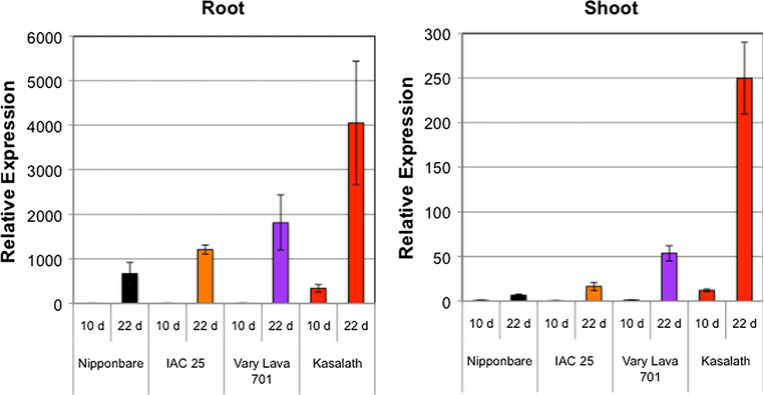
qRT-PCR analysis of *IPS1* in root and shoot of the 4 rice cultivars under Pi starvation. Both root and shoot samples showed significant upregulation after 10 and 22 days of growth in Pi deficient medium as compared to the control (0 d). The data represents the mean relative expression values (mean ± SE) of three technical replicates from one of the three biological replicates from each treatment used for RNA-Seq analysis

To correlate the morphological changes induced by −P with the molecular changes, the expression level of *IPS1* gene was analyzed by qRT-PCR. In both roots and shoots, the expression of *IPS1* was almost undetectable before treatment (0 d) and even until 10 days in Pi deficient medium (Fig. 1). However at 22 d of growth under Pi starvation, *IPS1* was upregulated more than 6× and 38× in Kasalath root and shoot, respectively, as compared to Nipponbare. Similarly, among the *japonica* cultivars, the expression levels of *IPS1* in IAC 25 and Vary Lava 701 were several folds higher than Nipponbare (Supplementary Fig. S4). This was also confirmed for other known Pi-upregulated genes such as *SQD2*, *SPX1*, *RNS3* and *PAP2* (Supplementary Fig. S4). Among them, acid phosphatase (PAP2) and RNase (RNS3) may be involved in the release of phosphate from cellular and extracellular organic compounds to facilitate more efficient P utilization. All relative inorganic P content in tolerant cultivars were lower than Nipponbare, except 22 d in root of IAC 25A (Supplementary Table S3). More upregulation of these genes in IAC 25, Vary Lava 701 and Kasalath as compared to Nipponbare was associated with lower relative inorganic P content which is an indicator of P utilization efficiency (Supplementary Table S3). Based on qRT-PCR analysis of *IPS1* and other well-known Pi starvation genes at 0 d (time of transfer) and 22 d in +P (normal medium), we confirmed that plant age did not affect the expression of these upregulated genes (Supplementary Fig. S4).

### RNA-Seq data sets and characterization of responsive transcripts

**Fig. 2:**
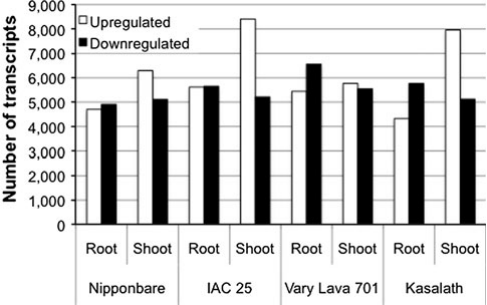
Distribution of responsive transcripts of the 4 rice cultivars under Pi starvation. The upregulated and downregulated transcripts were identified from RNA-Seq profiles of root and shoot after 22 days of growth in −P medium. These transcripts were aligned to the annotated genes in the IRGSP-1.0 reference genome as described in RAP-DB

We constructed a total of 48 libraries corresponding to root and shoot of the 4 cultivars for control (0 d) and −P treatment (22 d) with three biological replicates for root and shoot samples in each cultivar. The sequence reads generated were pre-processed and mapped onto the IRGSP-1.0 pseudomolecules (Table 2). For each cultivar, an average of approximately 21 million (92 %) reads were mapped to the reference genome sequence. The number of reads mapped to single locations (exonic regions and spliced-junctions) and multiple locations in the genome did not differ between the *japonica* and *indica* cultivars. On the other hand, an average 7.0 % of Nipponbare, 6.9 % of IAC 25, 6.0 % of Vary Lava 701 and 9.9 % of Kasalath quality-evaluated reads could not be aligned to the Nipponbare reference genome sequence. Among the mapped sequence reads, an average of about 5,000 reads from each cultivar that correspond to annotated transcripts in rice were found to be responsive under −P (Fig. 2). The number of upregulated transcripts ranged from 4,713 to 8,410 whereas the number of downregulated transcripts ranged from 4,909 to 6,561 among the 4 rice cultivars. The largest number of upregulated transcripts was obtained from shoots of IAC 25 (8,410 transcripts) and Kasalath (7,963 transcripts). Although the root of Vary Lava 701 showed relatively higher number of downregulated transcripts (6,561), there was not much difference in the number of responsive transcripts among the other 3 cultivars as well as the number of transcripts from shoot of the 4 cultivars.

**Table 2:** Mapping of RNA-Seq reads obtained from root and shoot samples of the 4 rice cultivars into the IRGSP-1.0 reference genome sequence

RNA-Seq library	Pre-processed	Aligned				Unaligned	
		Exonic regions	Spliced-junctions	Multi	(%)	Unaligned	(%)
Nipponbare							
Root_0 d	6,710,131	4,475,011	1,373,919	211,168	90.7	650,033	9.3
Root_22 d	7,583,250	4,812,812	1,458,180	233,711	85.9	1,078,548	14.1
Shoot_0 d	4,716,089	3,429,836	1,031,024	154,592	97.9	100,637	2.1
Shoot_22 d	5,902,794	4,317,447	1,246,361	194,275	97.5	144,711	2.5
IAC 25							
Root_0 d	6,545,589	4,418,582	1,369,562	203,343	91.8	554,103	8.2
Root_22 d	6,494,738	4,340,875	1,289,661	192,655	89.7	671,547	10.3
Shoot_0 d	4,819,847	3,347,756	1,020,824	232,480	95.4	218,788	4.6
Shoot_22 d	5,795,477	4,146,992	1,191,981	195,916	95.5	260,588	4.5
Vary Lava 701							
Root_0 d	7,843,126	5,190,227	1,596,516	249,449	89.6	806,935	10.4
Root_22 d	7,289,134	4,734,622	1,464,353	207,317	87.9	882,843	12.1
Shoot_0 d	5,373,057	3,808,638	1,152,511	195,007	96.0	216,901	4.0
Shoot_22 d	5,116,172	3,502,189	1,015,641	374,424	95.6	223,918	4.4
Kasalath							
Root_0 d	4,944,856	3,232,453	1,004,716	169,749	89.0	537,938	11.0
Root_22 d	3,214,951	2,008,991	577,209	101,751	84.2	527,001	15.8
Shoot_0 d	4,545,800	3,120,805	959,661	208,960	94.4	256,375	5.6
Shoot_22 d	4,541,704	3,160,472	905,711	152,953	92.9	322,568	7.1

### Identification of core Pi starvation responsive transcripts

**Fig. 3:**
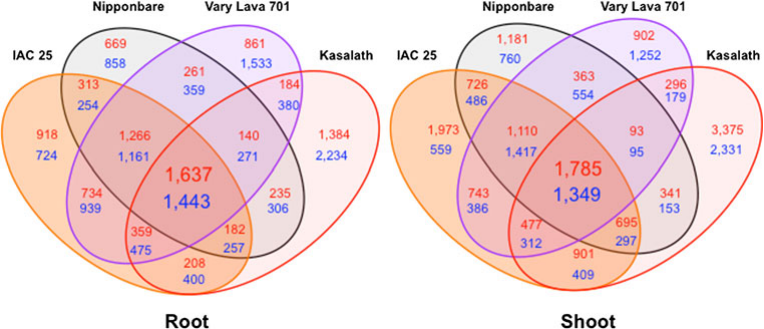
Venn diagram analysis of Pi starvation responsive transcripts. The resulting 4-way Venn diagrams for root and shoot show the number of upregulated (*red*) and downregulated (*blue*) genes after 22 d of growth in Pi deficient medium relative to the control (0 d)

The upregulated and downregulated transcripts with corresponding annotations were plotted in a Venn diagram (Fig. 3). As a result, we were able to identify −P responsive transcripts that overlapped among the different cultivars. Commonly upregulated or downregulated transcripts in the 4 cultivars could represent the core responsive genes under −P. A total of 1,637 transcripts in root and 1,785 transcripts in shoot were commonly upregulated whereas 1,443 transcripts in root and 1,349 transcripts in shoot were commonly downregulated among the 4 cultivars. The responsive transcripts in Nipponbare were used as reference for comparison of gene expression with the 3 other cultivars. Among 47 transcripts upregulated >10-fold in Nipponbare, a total of 42 transcripts were more upregulated in at least one of the three other cultivars (Supplementary Table S4). Similarly, among 56 transcripts downregulated <0.25 fold in Nipponbare, a total of 23 transcripts were less downregulated in at least one of the three other cultivars (Supplementary Table S5). The expression level in both IAC 25 and Vary Lava 701 was upregulated by as much as 5.5x as compared to Nipponbare. More than half of the same transcripts in Kasalath were more upregulated than the 3 *japonica* cultivars. The P1BS *cis*-acting element (GNATATNC), which regulates Pi-stress responsive transcripts upon binding with PHR1 transcription factor (Rubio et al. 2001), was confirmed in the 1 kb-upstream region from Nipponbare in 27.9 % (456 transcripts among 1,632 transcripts) and 25.0 % (446 transcripts among 1,783 transcripts) of core upregulated RAP-representative transcripts in root and shoot, respectively. In contrast 17.7 % (7,578 transcripts among 42,887 transcripts) and 17.8 % (7,588 transcripts among 42,736 transcripts) of the non-core RAP-representative transcripts. Moreover among these 456 and 446 transcripts containing P1BS, 96.5 % (440 transcripts) and 88.34 % (394 transcripts) were more upregulated in at least one cultivar among the three tolerant cultivars as compared to Nipponbare.

A total of 581 and 340 transcripts were commonly upregulated (Supplementary Table S6) and downregulated (Supplementary Table S7), respectively, in both root and shoot of the 4 cultivars. The upregulated transcripts include many Pi-related genes such as *IPS1*, *IPS2* (Wasaki et al. 2003b; Hou et al. 2005), *SPX1*, *SPX3* (Wang et al. 2009) and *ACP* (Bari et al. 2006). The downregulated transcripts include *PHO2/UBC24* (Bari et al. 2006). Transcripts with no distinct functions such as *Os12t0576600* (metallophosphoesterase domain containing protein), *Os02t0609000*, *Os02t0208500* (conserved hypothetical protein), *Os03t0603600* (PLC-like phosphodiesterase), *Os11t0658900* (similar to lipase family protein), *Os08t0280100* (similar to phytase), *Os01t0128200* (similar to nuclease I) and *Os04t0423400* (ABA/WDS induced protein) were also strongly upregulated. Strongly downregulated transcripts include *Os12t0274700* (petunia ribulose 1,5-bisphosphate carboxylase small subunit), *Os04t0380300* (kelch-type beta propeller domain containing protein), *Os05t0542200* (similar to catalytic/hydrolase), *Os11t0707000* (ribulose-bisphosphate carboxylase activase), *Os03t0689100* (histidine acid phosphatase family protein) *Os09t0246300* (conserved hypothetical protein), *Os05t0105800* (hypothetical protein) and *Os08t0157600* (MYB transcription factor). The expression of these genes were validated by qRT-PCR (Supplementary Fig. S5 and Supplementary Fig. S6). Although most of these genes have not been previously reported as Pi starvation responsive genes, a high level of expression in one or more cultivars may suggest specific functions associated with the response to Pi starvation.

**Fig. 4:**
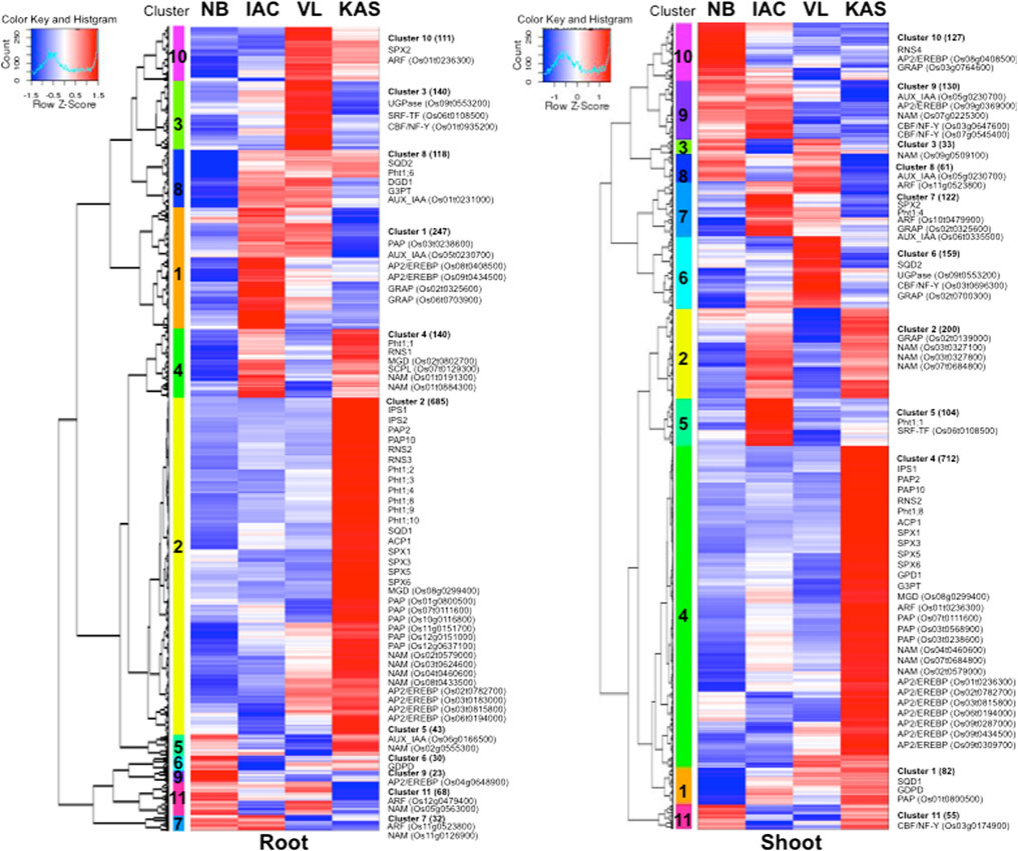
Hierarchical clustering of commonly upregulated transcripts in root and shoot of the 4 rice cultivars under Pi starvation. The z-score of fold-change values for each sample relative to the control (0 d) were subjected to hierarchical clustering using standard correlation. The average expression value for each transcript in the 4 cultivars was assigned a z-score = 0. The *color scale* in the histogram panel ranges from negative (*blue*) through neutral (*white*) to positive z-scores (*red*). The *number* and representative transcripts in each cluster are listed on the right. NB: Nipponbare, IAC: IAC 25; VL: Vary Lava 701; KAS: Kasalath

Hierarchical clustering (HCL) analysis of the commonly upregulated transcripts generated 11 clusters in root and 11 clusters in shoot with distinct gene expression patterns that distinguish the −P response of the 4 cultivars (Fig. 4). An overall view shows that the expression level of upregulated transcripts in Kasalath was relatively higher than the japonica cultivars. In particular, cluster 2 transcripts in root and cluster 4 transcripts in shoot were relatively more strongly upregulated in Kasalath as compared to the 3 other cultivars. These clusters consist of the major responsive transcripts under −P including genes associated with Pi stress such as *IPS1*, *PAP*, *NAM* etc. Among the 3 japonica cultivars, IAC 25 and Vary Lava 701 showed a higher proportion of upregulated transcripts than Nipponbare. Cluster 8 in root and cluster 1 in shoot consisted of transcripts that were predominantly more upregulated among the tolerant cultivars (IAC 25, Vary Lava 701 and Kasalath) as compared to Nipponbare. Transcripts upregulated in 2 tolerant genotypes include cluster 4 (IAC 25 and Kasalath) in root, cluster 10 (Vary Lava 701 and Kasalath) in root, and cluster 2 (IAC 25 and Kasalath) in shoot. Of the 11 distinct clusters in shoot, upregulated transcripts in specific genotypes were observed for cluster 4 (Kasalath), cluster 5 (IAC 25) and cluster 6 (Vary Lava 701). Transcripts upregulated in 2 tolerant genotypes include cluster 2 (IAC 25 and Kasalath), and cluster 7 (IAC 25 and Vary Lava 701). Most transcripts characterized in clusters 1, 2, 4, 5, 6 and 7 may function in shoot of rice under Pi starvation.

We performed GO enrichment analysis of upregulated and downregulated transcripts in shoot and root for transcripts using GO terms in the biological process category (Supplementary Fig. S7). Enriched GO terms significantly upregulated or downregulated in all 4 cultivars may represent the core responsive transcripts in rice under −P. Twelve GO terms (ex. phosphate ion transport [GO:0006817] and glycolysis [GO:0006096]) were associated with upregulated transcripts and two GO terms (transmembrane transport [GO:0055085] and nitrogen compound metabolic process [GO:0006807]) were associated with downregulated transcripts in both root and shoot under −P. Interestingly, malate metabolic process (GO:0006108), l-phenylalanine catabolic process (GO:0006559) and flavonoid biosynthetic process (GO:0009813) were enriched in upregulated transcripts of root and downregulated transcripts of shoot. Similarly, GO terms significantly upregulated or downregulated in IAC 25, Vary Lava 701 and Kasalath may represent specific transcripts that function mainly in −P tolerant cultivars. These include transcripts for response to oxidative stress (GO:0006979) and negative regulation of apoptotic process (GO:0043066) among significantly enriched upregulated transcripts. On the other hand, transcripts for ATP biosynthetic process (GO:0006754), ATP catabolic process (GO:0006200), mannose metabolic process (GO:0006013), carbon fixation (GO:0015977), intracellular protein transport (GO:0006886) and vesicle-mediated transport (GO:0016192) were among the significantly downregulated transcripts.

### Genotype specific Pi starvation responsive transcripts

Both the clustering analysis and GO enrichment analysis revealed genotype specificity of response to −P. In addition to cluster 2 transcripts in root and cluster 4 transcripts in shoot, which were significantly more upregulated in Kasalath, other clusters were also more significantly upregulated in specific genotypes. These include cluster 10 in Nipponbare shoot, cluster 5 in IAC 25 shoot and cluster 6 in Vary Lava 701 shoot (Fig. 4). Similarly, GO enrichment analysis also revealed genotype specific enriched GO terms. In Nipponbare, GO for glycolysis (GO:0006096) and defence response (GO:0006952) were enriched among the downregulated transcripts in root. In IAC 25, enriched GO for transport such as intracellular protein transport (GO:0006886) and vesicle-mediated transport (GO:0016192) among upregulated transcripts in shoot may be related to the internal translation of P and maintenance of growth activity. In Vary Lava 701, GO for tricarboxylic acid (TCA) cycle (GO:0006099), ATP hydrolysis coupled proton transport (GO:0015991), and ATP metabolic process (GO:0046034), were enriched among upregulated transcripts in root. In the tolerant cultivar Zhongzao 18, Li et al. (2010) reported that upregulation of several genes involved in the tricarboxylic acid cycle can improve the efficiency of Pi absorption under −P to produce more organic acids which are eventually released into the soil to activate the insoluble P. Kasalath showed the most number of specifically responsive transcripts. Significantly enriched GO terms include dephosphorylation (GO:0016311) and protein dephosphorylation (GO:0006470) that may function in Pi remobilization to enhance utilization efficiency in root. The GO terms for photosynthesis light harvesting, protein folding, translational elongation, translation, protein polymerization, DNA-dependent DNA replication initiation and DNA replication were enriched among downregulated transcripts in shoot resulting in growth retardation (Supplementary Fig. S2) and the repression of the synthesis of nucleic acids and proteins required for photosynthesis under −P.

### Identification of Pi starvation responsive unannotated transcripts

**Fig. 5:**
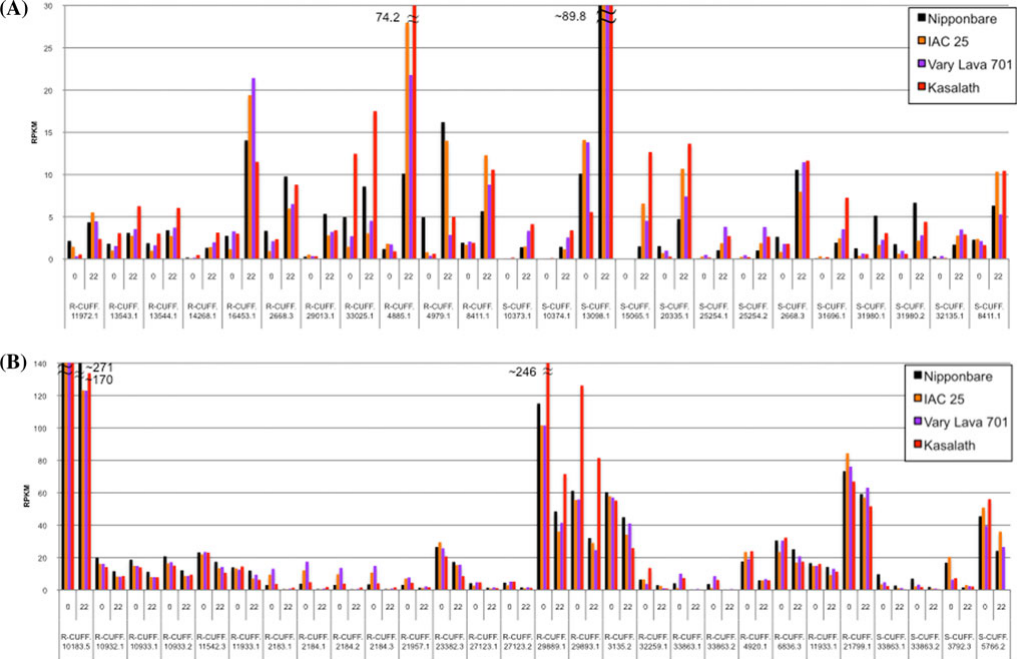
Expression value (RPKM) of unannotated transcripts commonly expressed in the 4 rice cultivars at 0 and 22 d of growth under Pi starvation. The RPKM values of upregulated (**a**) and downregulated (**b**) transcripts were identified by G-test (FDR < 0.01). Details for each transcript are described in Supplementary Table S8

We identified a total of 8,198 unique responsive transcripts from the root and shoot with no corresponding annotations in RAP-DB. A Blastx homology search resulted in 2,385 transcripts with homology to various proteins. We performed the G-test on the RPKM-derived read counts to determine the differences in gene expression in each genotype under −P and identified 24 commonly upregulated and 28 commonly downregulated transcripts in either root or shoot of the 4 cultivars (Fig. 5). Some transcripts such as R-CUFF.4885.1 and R-CUFF.13098.1 showed more than 4-fold increase in expression in all 4 cultivars under −P. These commonly responsive unannotated transcripts may be conserved among the 4 cultivars. Although some transcripts had much lower expression levels that could not be detected by statistical tests, the differences in expression level among the 4 cultivars were quite evident. The chromosome position and protein homology search results of these unannotated transcripts are shown in Supplementary Table S8. Among them, 14 transcripts showed high homology to known proteins. Transcripts without homology to any protein may include non-protein coding transcripts, novel protein transcripts, rare transcripts that are expressed at low copies, transcripts with very low expression levels, or even transcripts which may have lethal functions in *E. coli*.

### Characterization of unaligned reads expressed under Pi starvation

An average 7.4 % of the total sequence reads from each cultivar could not be mapped to the IRGSP-1.0 genome sequence. Although most of these unaligned reads may include artifacts such as low-quality reads, sequencing errors, or sequences derived from adaptors and contaminating organisms (Oono et al. 2011), some may also represent novel transcripts that may be involved in Pi starvation. De novo transcript assembly of these unaligned reads resulted in 33,078 Nipponbare contigs, 15,971 IAC 25 contigs, 23,707 Vary Lava 701 contigs and 13,994 Kasalath contigs with average length of 484 bp. Redundant contigs among the 4 cultivars comprising 90 % of total as well as unaligned contigs from Nipponbare were presumed to be artifacts and were removed. The unaligned reads were then used for alignment to the remaining contigs from IAC 25, Vary Lava 701 and Kasalath using bowtie. To characterize these contigs more accurately, we calculated the RPKM value for each assembly and performed G-test between the control and −P treatment. As a result, we identified 144 contigs from IAC, 194 contigs from Vary Lava 701, and 162 contigs from Kasalath in either root or shoot, which were responsive under −P (Supplementary Tables S9, S10). BLASTX search in RefSeq and Swissprot databases showed homology to amino acid sequences in rice as well as other organisms. We searched the *Pstol1* transcripts at *Pup1* locus (Gamuyao et al. 2012) conferring the tolerance among our contigs and found full-length transcripts in Kasalath as well as the japonica cultivars IAC 25 and Vary Lava 701 (identity 100 %, coverage 100 %). The transcript showed weak upregulation in the three tolerant genotypes. This result indicates that a similar allelic composition of the *Pup1* locus of IAC 25, Vary Lava and Kasalath (Chin et al. 2011), and further suggests that the contigs obtained from the different genotypes can be used for identification of genotype specificity. We also found that *HOX1* (Scarpella et al. 2005), a positive regulator of root cell differentiation was upregulated in root of Kasalath and IAC 25. Additionally, *DOS* (Kong et al. 2006), which was shown to delay leaf senescence in rice, was also upregulated in root of Kasalath. The contigs that changed to >100-fold and <0.1-fold under −P are shown in Supplementary Tables S9 and S10, respectively. Most of the upregulated contigs were obtained from Vary Lava 701 and included transcripts associated with −P response such as inorganic pyrophosphatase 1, nucleotide pyrophosphatase/phosphodiesterase, pyrophosphate-energized vacuolar membrane proton pump, protein-tyrosine phosphatase. Most of the downregulated contigs were also obtained from Vary Lava 701. These contigs which were not identified in Nipponbare may be specifically transcribed and function only in tolerant genotypes under −P.

## Discussion

### Identification of basal responsive transcripts under Pi starvation in rice

We investigated the dynamic expression patterns under −P treatment by identifying genes showing differential expression in the 4 rice cultivars using G-test (FDR < 0.01). Overall, approximately 20,030 (38.1 %) of 52,640 RAP-annotated transcripts showed significantly variable expression under −P treatment in at least one cultivar. This suggests that Pi starvation induce a marked systemic effect on the transcriptome of rice. Based on comparative analysis of the responsive transcripts among the 4 rice cultivars under −P, we were able to identify approximately 1,500 annotated transcripts, including many well-known Pi related genes, and several unannotated transcripts as core responsive transcripts (Figs. 3, 4, 5). Several upregulated and downregulated core genes in both root and shoot were validated by qRT-PCR (Supplementary Figs. S5, S6). We used the public microarray data (GSE6901, http://www.ncbi.nlm.nih.gov/geo/) to compare the expression of these genes to other abiotic stresses. At 2-fold or 0.5-fold cut-off and 10-fold or 0.1-fold cut-off, less than 20 % and 2 % core responsive genes, respectively, were also responsive to drought, salt and cold stress. This suggests that a large proportion of core responsive genes identified in this study may be specifically expressed in response to −P. Most of the upregulated transcripts were more strongly expressed in the tolerant *indica* cultivar Kasalath as well as *japonica* cultivars IAC 25 and Vary Lava 701 with relatively higher tolerance to −P stress than Nipponbare (Fig. 4). Existing substantial expression diversity in the core transcripts should account for the difference in response to −P between the subspecies *japonica* and *indica* as well as among the *japonica* cultivars. Furthermore, we have identified core transcripts expressed in both root and shoot as well as other tissue-specific core transcripts. Thus, RNA-Seq accurately measures the expression frequencies of genes over a broad dynamic range and detects previously annotated as well as unannotated transcripts that are not supported by the microarray platform in rice. For overall gene expression, we observed high correlation coefficient, suggesting a clear validation of the microarray-based gene expression profiling data with the RNA-Seq data (Oono et al. 2011). In addition, we were able to identify transcripts from IAC 25, Vary Lava 701 and Kasalath which could not be aligned to the Nipponbare genome sequence. The RNA-Seq could therefore be an efficient strategy in identifying novel transcripts particularly in cultivars with no genome sequence information.

### Genotypic variation in P content, P utilization and biomass under Pi starvation

Substantial expression diversity among the 4 cultivars also exists in non-core responsive transcripts. In general, Kasalath showed a higher percentage of annotated −P responsive transcripts than those obtained from the other cultivars (Fig. 3). In all analyses, diversity in expression level was most prominent in Kasalath among the 4 cultivars. In general, *indica* cultivars have been shown to maintain higher relative P content and can be classified as more tolerant to −P than *japonica* cultivars in Pi deficient soil (Wissuwa and Ae 2001). Using P content as a measure of acquisition efficiency, IAC 25, Vary Lava 701 and Kasalath showed higher relative total P content in the roots indicating a more efficient P acquisition as compared to Nipponbare. In shoot however, Vary Lava 701 showed the highest P content among the 4 cultivars. These genotypic variations in morphology and physiological processes associated with root and shoot growth could be adaptive measures of each cultivar to enhance P acquisition under starvation. To understand the P utilization efficiency of each cultivar, we investigated the effect of −P stress treatment on inorganic P content (Supplementary Table S3). The inorganic P content relative to control was lower in tolerant cultivars as compared to Nipponbare at 10 and 22 d, except in root of IAC 25 at 22 d suggesting that tolerant cultivars tend to reduce inorganic P content to facilitate more efficient P utilization. The P utilization efficiency in root of Kasalath might have been enhanced after 22 d under −P as shown by upregulation of several genes associated to Pi starvation response (Supplementary Fig. S4) and enrichment of GO terms for dephosphorylation and protein dephosphorylation (Supplementary Fig. S7).

With an increase in root weight of Kasalath under −P, the ratio of shoot weight to root weight was decreased. The root system may have been modified to maximize Pi interception, solubilisation and acquisition under −P, the efficiency of which might be affected by the developmental stage, growth condition, and treatment. Based on this observation, it can be assumed that P acquisition from root is more important for maintaining homeostasis. Both P acquisition efficiency and P utilization efficiency are important for characterization of genotype under −P. In shoot of Kasalath, the GO terms for photosynthesis, light harvesting, translation and DNA replication were enriched among downregulated transcripts, and could therefore be correlated with growth inhibition (Supplementary Figure S1, S2). In root, P content of IAC 25 and Kasalath was higher than Nipponbare but P concentration was lower than Nipponbare (Table 1). Both P content and P concentration of Vary Lava 701 in root were higher than Nipponbare (Table 1). Although both cultivars are more tolerant under −P, a large portion of responsive transcripts as well as their expression levels also showed variation among these cultivars (Figs. 3, 4). These results indicate significant variations in P content and gene expression among the 4 cultivars. However, the regulation of these parameters is quite complex and would require more detailed analysis.

### Genotype specific transcripts for tolerance to Pi starvation

**Fig. 6:**
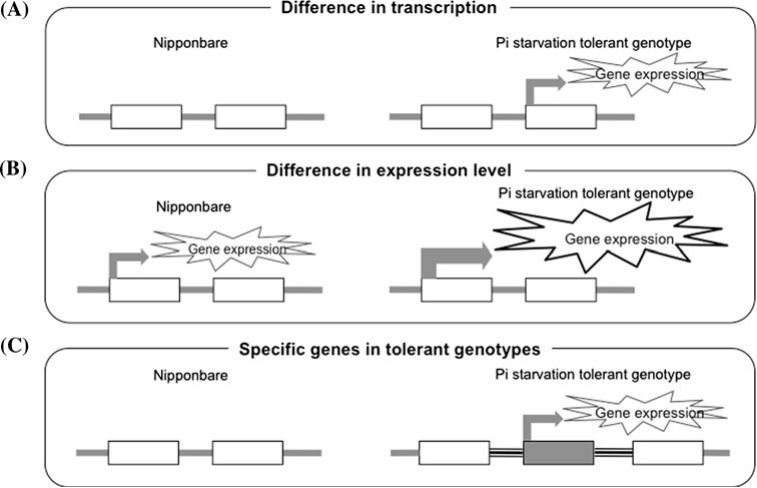
Model systems of gene expression in rice under Pi starvation as revealed by RNA-Seq analysis of rice cultivars with different levels of tolerance. Three model systems in reference to the Nipponbare gene expression profile have been clarified based on **a** difference in transcription among the 4 genotypes, **b** difference in expression level of transcripts among the 4 genotypes, and **c** difference of expressed transcripts between Nipponbare and Pi starvation tolerant genotypes

The expression profiles of the 4 rice cultivars revealed by RNA-Seq provided not only an overview of the diversity in rice transcriptome under −P but also reflect inherent strategies among these cultivars in overcoming stress due to −P. Comparison of the transcriptome of Nipponbare under −P with more tolerant *japonica* cultivars (IAC 25 and Vary Lava 701) and the *indica* cultivar Kasalath revealed a diversity in the transcriptome that reflects various degrees of tolerance of different genotypes. The difference in transcriptomes among the 4 genotypes may be associated with transcripts responsive in tolerant cultivars but totally unresponsive in Nipponbare (Fig. 6a). These include NAM (NAC) transcription factor genes such as *Os01t0191300*, *Os04t0619000*, *Os07t0684800* and *Os12t0477400* which showed variation in response to −P among the tolerant cultivars. It has been reported that NAC1 mediated auxin signalling whereas NAC2 mediated auxin and ethylene signalling that promote lateral root development (Xie et al. 2000; He et al. 2005). In *Arabidopsis*, several members of Class IIB NAC transcription factor family were found to regulate cellular differentiation and cell wall modification associated with root cap maturation (Bennett et al. 2010). The changes in root development in response to −P have also been widely documented. Early root growth is enhanced by *PSTOL1* thereby enabling the rice plant to acquire more phosphorus and other nutrients from the soil (Gamuyao et al. 2012). Overexpression of *OsMYB2P*-*1* in rice enhanced tolerance to −P and the development of longer primary and adventitious roots under −P (Dai et al. 2012). Overexpression of *PHR2* (substitute for *PHR1* in case of rice) in the PHR1-miR399-IPS1-PHO2/UBC24 pathway which is a central component of the Pi starvation was found to mimic −P stress in rice with enhanced root elongation and proliferated root hair growth (Zhou et al. 2008). Here we found that *PHR2* was not strongly responsive under −P as previously reported (Zhou et al. 2008). In addition, we also found that upregulated transcripts have tendency to be more upregulated in tolerant genotypes (Fig. 4). This may have resulted from a more extensive root system in tolerant genotype than non-tolerant genotype and an enhanced signal transduction resulting in alteration of root architecture. *AUX/IAA* (*Os01t0231000, Os03t0742900)* and *AP2/EREBP* (*Os10g0390800*) were also upregulated in root under −P stress. Stable expression of AtPUCHI, one of AP2/EREBP transcription factors required auxin-responsive elements in its promoter region, contributes to lateral root morphogenesis by affecting the pattern of cell divisions during the early stages of primordium development (Hirota et al. 2007). *WRKY* (*Os01t0734000*) was also upregulated in root under −P stress. In *Arabidopsis*, WRKY6 and WRKY42 modulate PHO1 transcription (Chen et al. 2009) whereas WRKY75 modulates Pi acquisition and root development (Devaiah et al. 2007). Specific transcripts in tolerant genotypes may also function as specific tolerance strategy in each genotype as shown by GO enrichment analysis (Supplementary Fig. S7).

### Role of P1BS *cis*-acting element in Pi starvation signalling

The difference in transcriptome between Nipponbare and tolerant cultivars maybe associated with the level of expression among the core transcripts that were generally responsive under Pi starvation stress including many known stress related responsive transcripts. We found that most upregulated transcripts have tendency to be upregulated more in tolerant genotypes as compared with Nipponbare (Fig. 6b). However, further verification such as overexpression of some genes in Nipponbare will be necessary to establish the relationship between tolerance and transcript level. Several genes showed higher expression in roots of tolerant cultivars putatively associated with root cell wall loosening and root hair extension (Pariasca-Tanaka et al. 2009) and glycolysis and TCA cycle (Li et al. 2010) under −P. Overexpression of *OsMYB2P*-*1* in rice enhanced tolerance to −P with greater expression of −P responsive genes such as *OsIPS1, OsPAP10* and several high-affinity Pi transporters (Dai et al. 2012). In total, 27.9 % (456 transcripts) of core upregulated transcripts in root and 25.0 % (446 transcripts) of core upregulated transcripts in shoot of Nipponbare have P1BS in their promoter region. Moreover, among these P1BS-containing transcripts, 96.5 % (440 transcripts) in root and 88.3 % (394 transcripts) in shoot were more upregulated in at least one of the three tolerant cultivars as compared to Nipponbare. These suggest that a Pi-signalling mediated major system PHR1-IPS1-miR399-PHO2/UBC24 (Bari et al. 2006) and P1BS may enhance the expression of core responsive transcripts under Pi starvation. Bustos et al. (2010) analyzed the P1BS representation relative to the x-fold induction and showed a striking correlation between inducibility and P1BS content only in the 1 kb promoter regions. In addition to the P1BS system, there maybe other systems that mediate stress tolerance in rice under Pi starvation.

### Changes in gene expression associated with genomic structure

The differences among cultivars in response to −P may be attributed to the differences in the genomic structure of transcripts as well as the differences in expression level. Although −P responsive transcripts derived from IAC 25, Vary Lava 701 and Kasalath were mapped onto the Nipponbare genome, the promoter regions may differ among the genotypes resulting in variation in the control of transcription and response to Pi starvation. One possible inherent factor is DNA methylation and histone modifications in the transcribed region of responsive genes. A chromatin-level regulation of −P response genes that involved the deposition of histone H2A.Z and resulting in multiple phenotypes has been demonstrated in *Arabidopsis* (Smith et al. 2010). Furthermore, differential epigenetic modifications that have been correlated with changes in transcript levels among hybrids and parental lines based on analysis of single nucleotide polymorphisms (SNP) of the genome sequence (He et al. 2010) could also account for the differences in response to −P. The SNP in the regulatory region of specific genes has been found to induce significant alteration of gene expression as reported in the loss of seed shattering in Nipponbare owing to the absence of abscission layer formation (Konishi et al. 2006). A single mutation that resulted in a frame-shift deletion within the *Rc* gene was known to induce the change in seed colour from red in wild rice to white in cultivated rice (Sweeney et al. 2007). Furthermore, minor changes in sequence during domestication of cultivated rice have also been associated with genes such as *Bh4* (hull colour), *PROG1* (tiller angle), *sh4* (seed shattering), *qSW5* (grain width) and *OsC1* (leaf sheath colour and apiculus colour) (Huang et al. 2012). The evolution of morphological features has been associated with changes in the cis-regulatory sequences as induced by various biochemically functional elements and buffering action of enhancers (Meireles-Filho and Stark 2009). In the present study, various novel responsive transcripts from the 4 cultivars identified among unaligned reads (Supplementary Tables S9, S10) also suggest differences in genomic structure associated with the response to Pi-starvation. Transposon-mediated transcriptional control of neighbouring genes may also add to the complexity of the regulatory networks that can be initiated by transposon insertions that render adjacent genes stress-inducible (Naito et al. 2009). Therefore, in the process of hybridization to develop new cultivars, epigenetic modifications may have occurred resulting in differences in expression, protein activity, and target specificity from −P tolerance.

In the case of adaptation to Pi stress, the difference in response between Nipponbare and the three tolerant cultivars could be possibly associated to orthologous genes that evolved from a common ancestral gene but eventually diverged in structure and function to a certain degree. Therefore the difference in transcriptome among genotypes could also be associated to the presence of responsive transcripts totally absent in Nipponbare (Fig. 6c). These responsive transcripts include sequence reads that could not be mapped to the Nipponbare genome but showed homology to known sequences (Supplementary Tables S9, S10). In the case of *Pup1*, a major −P tolerance QTL located on rice chromosome 12 was initially identified in Kasalath and molecular markers evenly distributed over the fine-mapped 278-kb *Pup1* region were found to differ in allele constitutions in 81 rice accessions (Chin et al. 2011). This may suggest that other −P responsive genes in the three tolerant cultivars may have alleles that are totally undetected in Nipponbare. We searched the *Pstol1* (Gamuyao et al. 2012) among unaligned contigs and found full-length transcripts in Kasalath as well as the *japonica* cultivars IAC 25 and Vary Lava 701. Overall, we obtained a few thousand contigs from these unaligned reads in the Nipponbare genome. Homology search of these contigs revealed a wide range of possible putative functions that maybe directly or indirectly involved in response to −P among different rice cultivars. These contigs may represent genes involved in the biochemical adaptation of Pi-starved plants. The genetic basis of specific differences between Nipponbare and tolerant genotypes must be explored further based on the expression patterns, distribution of reads, and responsive contigs to elucidate the mechanisms involved in tolerance to −P. Recent studies have also shown that OsPHF1 (Pi transporter traffic facilitator) was involved in trafficking Pi transporters from endoplasmic reticulum to plasma membrane that resulted in adjustment of Pi uptake ability (Wu et al. 2013, Chen et al. 2011). Thus, in addition to transcriptional level, it would necessary to elucidate posttranscriptional regulation mechanisms for more comprehensive understanding of tolerance under −P.

## Conclusion

In this study, we were able to characterize the diversity of transcriptomes under −P based on RNA-Seq profiles of 4 rice genotypes. Additionally, we were able to identify many annotated, unannotated and unaligned responsive transcripts for accessing, mobilization, acquisition and utilization of Pi under stress conditions. Variation in the expression of these transcripts provides an overall view on how genotypes with different levels of tolerance to Pi stress respond under −P. Genotypic differences in overcoming Pi stress could be associated with differences in the genomic structure of transcripts involved in tolerance to Pi stress, differences in expression level of core responsive transcripts, and genotype-specific genes that play significant roles in overcoming Pi stress. These results will be useful deciphering gene networks involved in −P stress and for identifying genes that could be exploited in breeding for P-efficient and high yielding cultivars under −P.

## Electronic supplementary material

Below is the link to the electronic supplementary material.

Supplementary material 1 (XLS 18 kb)

Supplementary material 2 (XLS 29 kb)

Supplementary material 3 (XLS 30 kb)

Supplementary material 4 (XLS 45 kb)

Supplementary material 5 (XLS 50 kb)

Supplementary material 6 (XLS 276 kb)

Supplementary material 7 (XLS 175 kb)

Supplementary material 8 (XLS 39 kb)

Supplementary material 9 (XLS 35 kb)

Supplementary material 10 (XLS 37 kb)

**Supplementary Fig. S1** Growth of various rice cultivars after 22 days in culture medium with +P (10 mg P/L; control) and culture medium with −P (0.1 mg P/L). (TIFF 2931 kb)

**Supplementary Fig. S2** Effect of phosphate starvation stress on biomass production. Changes in the shoot dry weight (**a**), root dry weight (**b**), and shoot/root dry weight ratio of the 4 cultivars under +P (0.323 mM NaH_2_PO_4_; control), −P (0.00323 mM NaH_2_PO_4_) and ++P (3.23 mM NaH_2_PO_4_) treatment conditions (**c**). The values represent the mean ± SE for three replicates for each treatment. Statistical significances of differential expression between treatments were tested by Student’s *t* test for the shoot/root dry weight ratio. The asterisks show statistical significances (*; P < 0.05 and **: P < 0.01). (TIFF 2931 kb)

**Supplementary Fig. S3** Effect of Pi starvation stress treatment on total P content and P concentration of rice seedlings in root and shoot at 0 day (control), 10 days and 22 days in +P (control) and −P culture medium. (TIFF 2931 kb)

**Supplementary Fig. S4** qRT-PCR analysis of *IPS1* and some well-known Pi starvation upregulated genes in root and shoot of the 4 rice cultivars after 22 days of +P and −P treatments. Both root and shoot samples showed significant higher upregulation after 22 days of growth in Pi deficient medium than it in +P (22 d), as compared to the control (0 d). The data represents the mean relative expression values (mean ± SE) of three technical replicates for each treatment. (TIFF 2931 kb)

**Supplementary Fig. S5** qRT-PCR analysis of upregulated core responsive genes in root and shoot of the 4 rice cultivars after 10 and 22 days of Pi starvation. Both root and shoot samples showed significant upregulation after 22 days of growth in Pi deficient medium as compared to the control (0 d). The data represents the mean relative expression values (mean ± SE) of three technical replicates for each treatment. (TIFF 2931 kb)

**Supplementary Fig. S6** qRT-PCR analysis of downregulated core responsive genes in root and shoot of the 4 rice cultivars after 10 and 22 days of Pi starvation. Both root and shoot samples showed significant downregulation after 22 days of growth in Pi deficient medium as compared to the control (0 d). The data represents the mean relative expression values (mean ± SE) of three technical replicates for each treatment. (TIFF 2931 kb)

**Supplementary Fig. S7** Identification of Gene Ontology (GO) terms of root and shoot transcripts enriched among the 4 rice cultivars in response to Pi starvation. Significant GO terms identified by GO enrichment analysis based on the most enriched biological processes associated with each cultivar under Pi starvation are shown in heatmap (−log10 of FDR values) for transcripts commonly expressed in the 4 cultivars. The bar in red–black gradation indicates the level of significance of GO enrichment with the extremes representing statistically significant (red) and not statistically significant (black) GO terms. Commonly enriched GO terms among all cultivars are represented in rows 1 and 7; among tolerant cultivars (IAC 25, Vary Lava 700, Kasalath) in rows 2 and 8; and the rest corresponds to enriched GO terms in each cultivar. The number of transcripts used for the GO analysis is indicated in the Venn diagram shown in Figure 3 **a** (root) and 3b (shoot). (TIFF 2931 kb)

Supplementary material 18 (TIFF 2931 kb)
