# Supplementary figures and images for: Diversity in the complexity of phosphate starvation transcriptomes among rice cultivars based on RNA-Seq profiles
Source: Plant Mol Biol. 2013 Jul 16;83(6):523–37. doi: 10.1007/s11103-013-0106-4 (PMC3830200; doi:10.1007/s11103-013-0106-4)

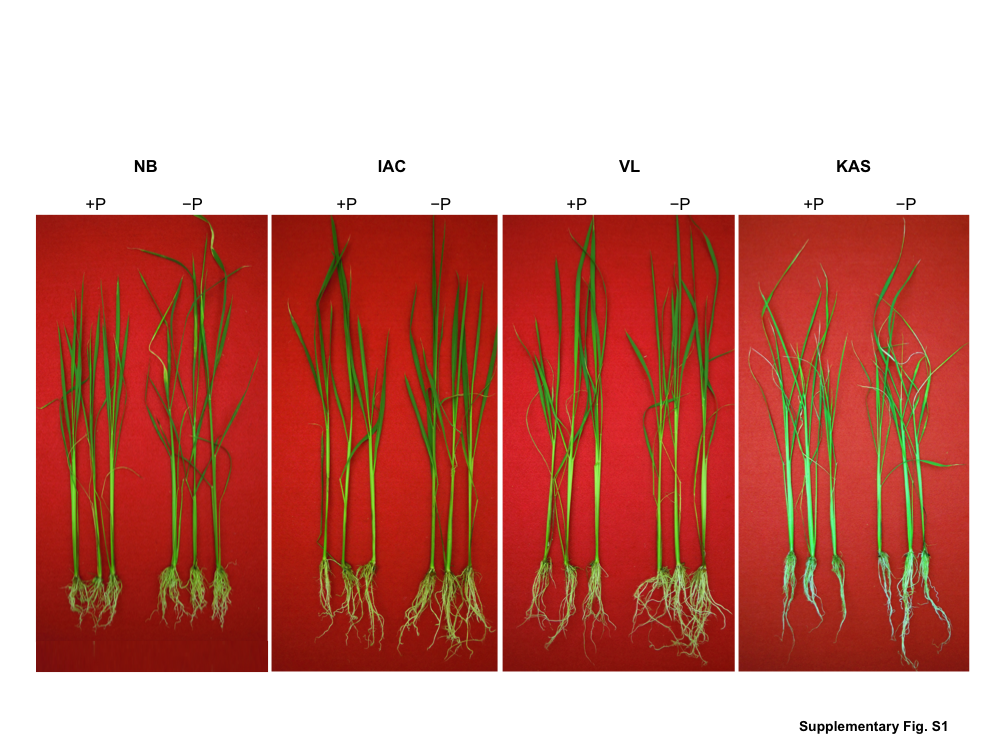

Supplement: Supplementary file 11 — Supplementary Fig. S1 Growth of various rice cultivars after 22 days in culture medium with +P (10 mg P/L; control) and culture medium with −P (0.1 mg P/L). (TIFF 2931 kb) [file 11103_2013_106_MOESM11_ESM.tif]

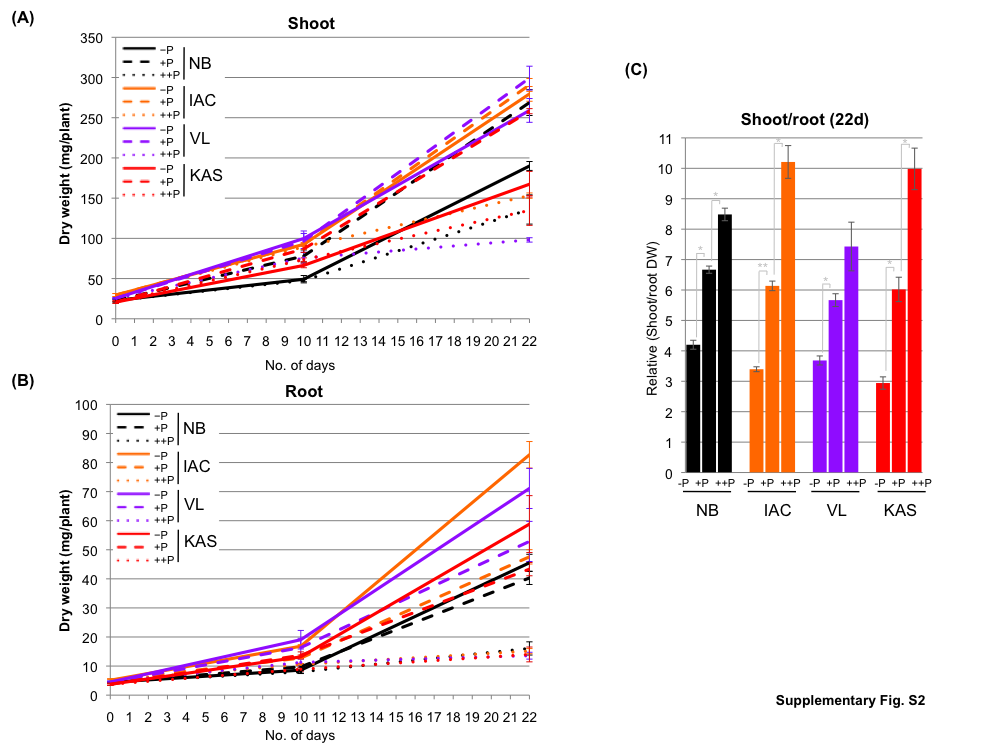

Supplement: Supplementary file 12 — Supplementary Fig. S2 Effect of phosphate starvation stress on biomass production. Changes in the shoot dry weight (a), root dry weight (b), and shoot/root dry weight ratio of the 4 cultivars under +P (0.323 mM NaH2PO4; control), −P (0.00323 mM NaH2PO4) and ++P (3.23 mM NaH2PO4) treatment conditions (c). The values represent the mean ± SE for three replicates for each treatment. Statistical significances of differential expression between treatments were tested by Student’s t test for the shoot/root dry weight ratio. The asterisks show statistical significances (*; P < 0.05 and **: P < 0.01). (TIFF 2931 kb) [file 11103_2013_106_MOESM12_ESM.tif]

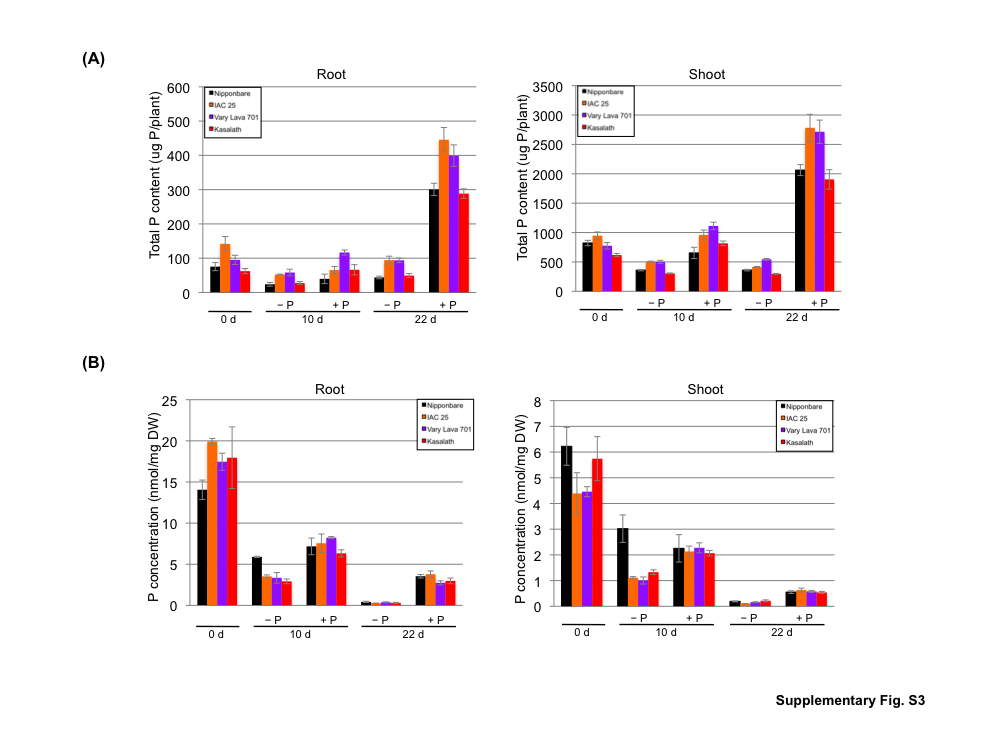

Supplement: Supplementary file 13 — Supplementary Fig. S3 Effect of Pi starvation stress treatment on total P content and P concentration of rice seedlings in root and shoot at 0 day (control), 10 days and 22 days in +P (control) and −P culture medium. (TIFF 2931 kb) [file 11103_2013_106_MOESM13_ESM.tif]

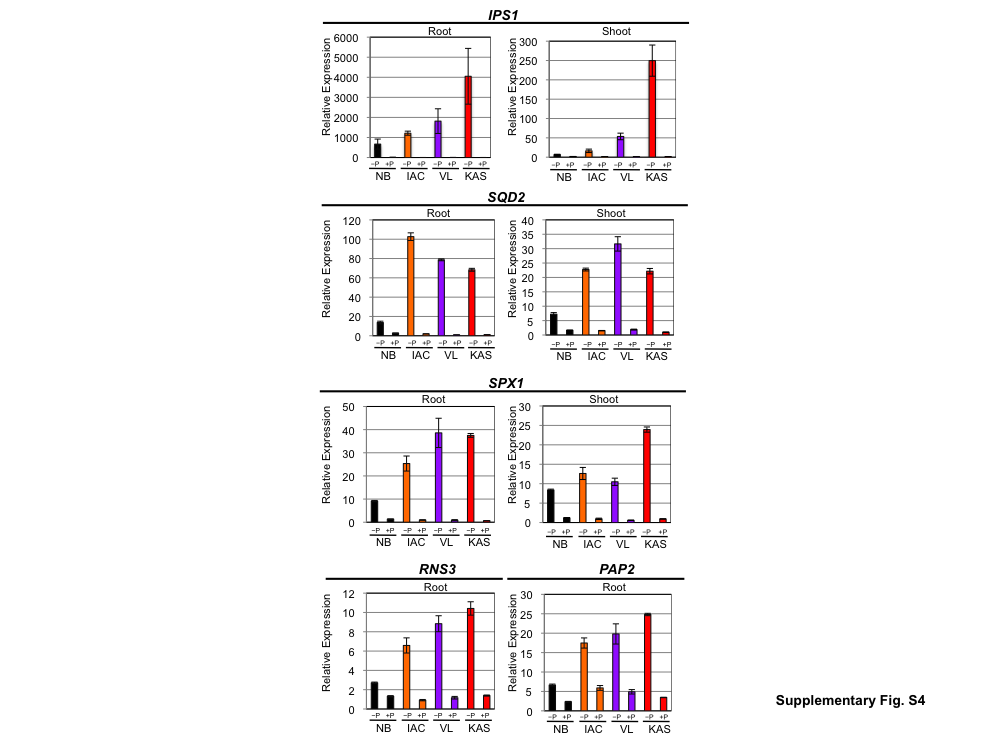

Supplement: Supplementary file 14 — Supplementary Fig. S4 qRT-PCR analysis of IPS1 and some well-known Pi starvation upregulated genes in root and shoot of the 4 rice cultivars after 22 days of +P and −P treatments. Both root and shoot samples showed significant higher upregulation after 22 days of growth in Pi deficient medium than it in +P (22 d), as compared to the control (0 d). The data represents the mean relative expression values (mean ± SE) of three technical replicates for each treatment. (TIFF 2931 kb) [file 11103_2013_106_MOESM14_ESM.tif]

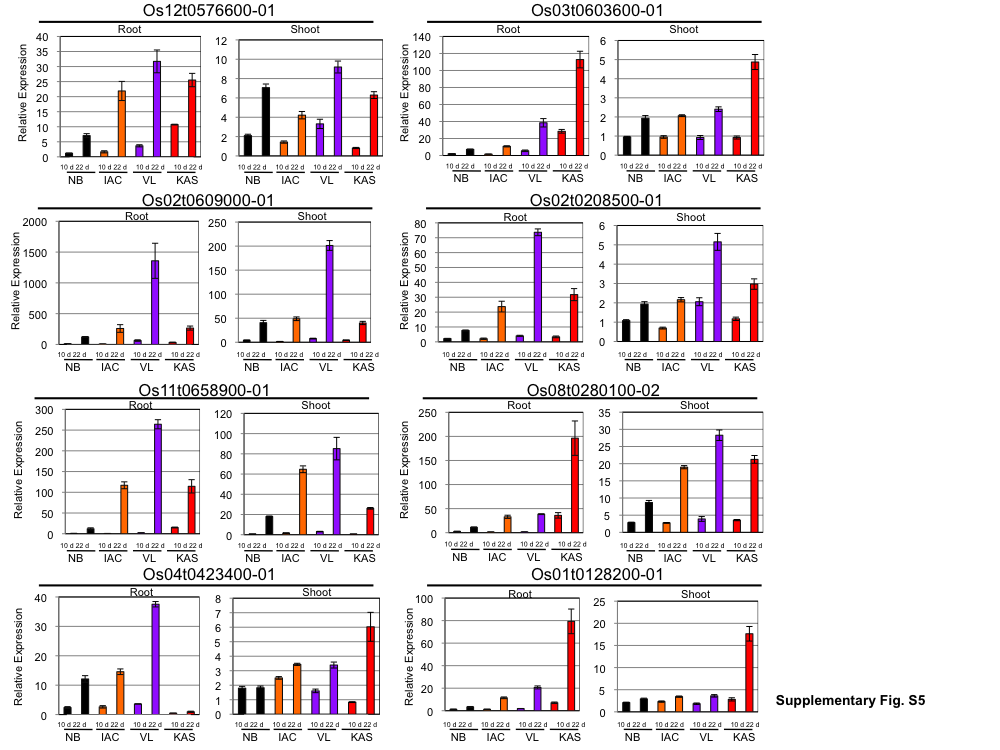

Supplement: Supplementary file 15 — Supplementary Fig. S5 qRT-PCR analysis of upregulated core responsive genes in root and shoot of the 4 rice cultivars after 10 and 22 days of Pi starvation. Both root and shoot samples showed significant upregulation after 22 days of growth in Pi deficient medium as compared to the control (0 d). The data represents the mean relative expression values (mean ± SE) of three technical replicates for each treatment. (TIFF 2931 kb) [file 11103_2013_106_MOESM15_ESM.tif]

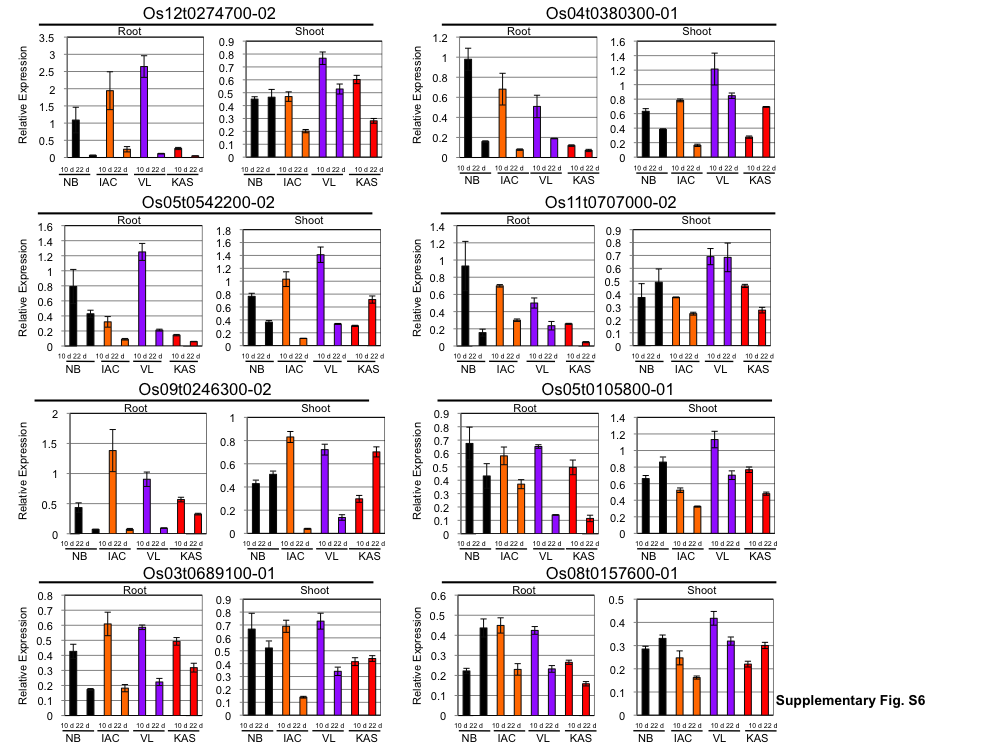

Supplement: Supplementary file 16 — Supplementary Fig. S6 qRT-PCR analysis of downregulated core responsive genes in root and shoot of the 4 rice cultivars after 10 and 22 days of Pi starvation. Both root and shoot samples showed significant downregulation after 22 days of growth in Pi deficient medium as compared to the control (0 d). The data represents the mean relative expression values (mean ± SE) of three technical replicates for each treatment. (TIFF 2931 kb) [file 11103_2013_106_MOESM16_ESM.tif]

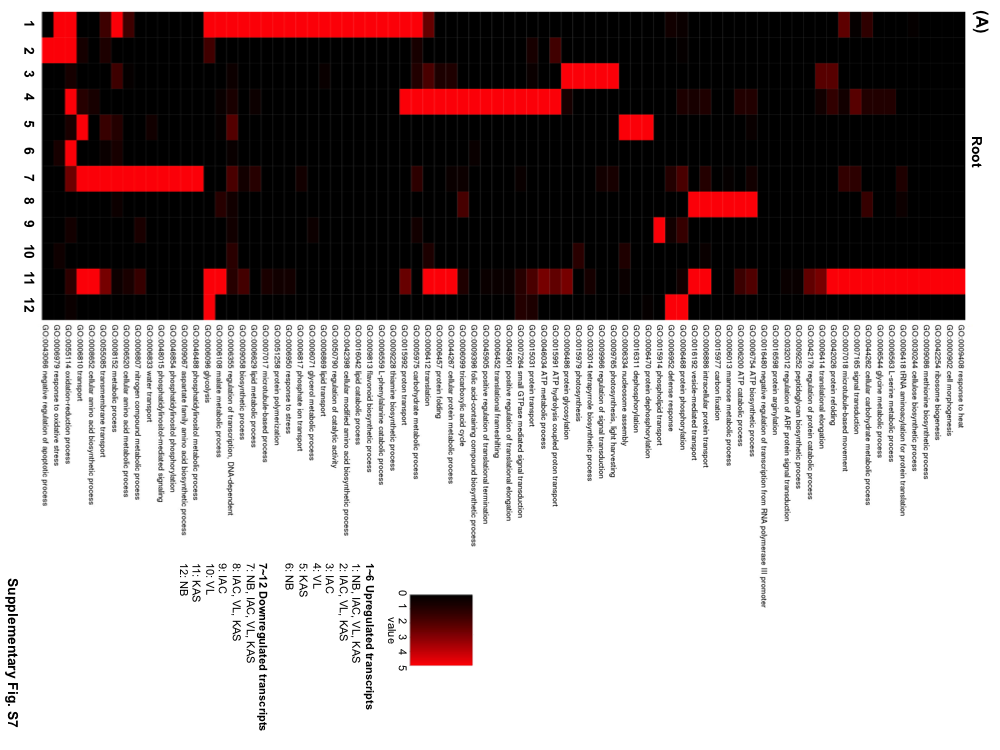

Supplement: Supplementary file 17 — Supplementary Fig. S7 Identification of Gene Ontology (GO) terms of root and shoot transcripts enriched among the 4 rice cultivars in response to Pi starvation. Significant GO terms identified by GO enrichment analysis based on the most enriched biological processes associated with each cultivar under Pi starvation are shown in heatmap (−log10 of FDR values) for transcripts commonly expressed in the 4 cultivars. The bar in red–black gradation indicates the level of significance of GO enrichment with the extremes representing statistically significant (red) and not statistically significant (black) GO terms. Commonly enriched GO terms among all cultivars are represented in rows 1 and 7; among tolerant cultivars (IAC 25, Vary Lava 700, Kasalath) in rows 2 and 8; and the rest corresponds to enriched GO terms in each cultivar. The number of transcripts used for the GO analysis is indicated in the Venn diagram shown in Figure 3 a (root) and 3b (shoot). (TIFF 2931 kb) [file 11103_2013_106_MOESM17_ESM.tif]

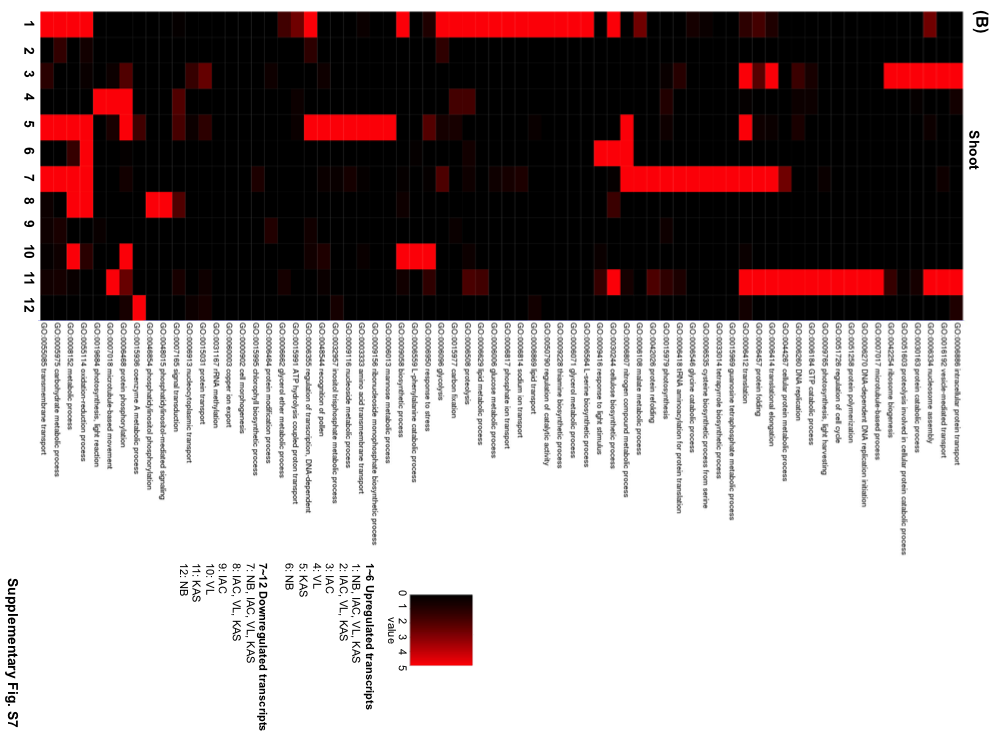

Supplement: Supplementary file 18 — Supplementary material 18 (TIFF 2931 kb) [file 11103_2013_106_MOESM18_ESM.tif]
